# Sonicated Extract from the Aril of *Momordica Cochinchinensis* Inhibits Cell Proliferation and Migration in Aggressive Prostate Cancer Cells

**DOI:** 10.1155/2022/1149856

**Published:** 2022-12-27

**Authors:** Seksom Chainumnim, Sunit Suksamrarn, Faongchat Jarintanan, Suchada Jongrungruangchok, Sivaporn Wannaiampikul, Wanlaya Tanechpongtamb

**Affiliations:** ^1^Department of Biochemistry, Faculty of Medicine, Srinakharinwirot University, Bangkok 10110, Thailand; ^2^Department of Chemistry, Center of Excellence for Innovation in Chemistry, Faculty of Science, Srinakharinwirot University, Bangkok 10110, Thailand; ^3^Faculty of Medical Technology, Rangsit University, Pathum Thani-12000, Thailand; ^4^Faculty of Pharmacy, Rangsit University, Pathum Thani-12000, Thailand

## Abstract

*Momordica cochinchinensis* or gac fruit has been reported to have several biological activities, including antioxidation, anti-inflammatory, and anticancer activities. However, the effect on cancer cell metastasis has not been extensively studied. With this aim, the extract from the aril part was selected and investigated for prostate cancer cell migration. The aril extracts were prepared as boiled extract, sonicated extract, ethanol extract, and HAE (hexane:acetone:ethanol; 2 : 1 : 1) extract, while the prostate cancer cell models were PC-3 and LNCaP cells. An MTT assay was performed to compare the antiproliferative effect between prostate cancer cells and normal Vero cells. As a result, the sonicated extract had the highest efficiency in PC-3 cells, with IC_50_ values of 2 mg/mL and 0.59 mg/mL for 48 and 72 h, respectively, while it had less of an effect in LNCaP cells and was not toxic to normal cells. Cell damage was further confirmed using LDH and cell cycle analysis. As a result, the sonicated extract did not cause cell damage or death and only inhibited cell proliferation. The effect on cancer metastasis was further examined by wound healing, transwell migration assays, and western blotting. The results demonstrated that the sonicated extract inhibited PC-3 cell migration and decreased MMP-9 but increased TIMP-1 expression. All these results support that gac fruit is a valuable source for further development as an anticancer agent for prostate cancer patients.

## 1. Introduction

Prostate cancer is a disease that occurs from the uncontrolled growth of abnormal cells in the prostate gland. The risk of prostate cancer increases with age, especially after age 50. It has been reported that prostate cancer is the second most common cancer that leads to mortality in older men. In Thailand (2020), 8,630 new prostate cancer cases (9.2%) and 3,837 prostate cancer-related deaths (3.1%) were found [1]. The treatment of prostate cancer can be performed using various methods, such as surgery, radiotherapy, chemotherapy, and hormonal therapy, depending on symptoms and cancer staging [2, 3]. New methods have also been proposed and applied for prostate cancer patients such as targeted therapy (Olaparib (Lynparza), Rucaparib (Rubraca)), immunotherapy (sipuleucel-T (Provenge), pembrolizumab), bone-modifying drugs (denosumab (Prolia, Xgeva), zoledronic acid (Reclast, Zometa), alendronate (Fosamax), risedronate (Actonel), ibandronate (Boniva), and pamidronate (Aredia)) [4–6]. However, all treatments are effective mostly for the early stage. The late or metastatic stage is still untreatable. Hence, a new therapeutic or drug that could reduce the progression to the metastatic stage may be helpful to enhance therapeutic efficiency in prostate cancer patients. Since natural resources are currently receiving much attention for anticancer drug development [7–9], natural extracts from plants were the focus of this work, and the mechanisms of cancer cell proliferation and metastasis suppression were targeted.

*Momordica cochinchinensis* (Lour.) Spreng or gac fruit was selected in the present work for its various biological activities. It is a vine plant of the Cucurbitaceae family found in Southeast Asia [10, 11]. Gac is widely used as a food, fruit juice drink, ingredient for cooking, and alternative medicine in Asian countries, including China, Vietnam, and Thailand [12]. Seeds, stems, and aril parts of gac have been shown to have valuable activities, including antioxidant, anti-inflammatory, and anticancer activities [13, 14]. The aril is the tissue surrounding the seed and contains a high level of carotenoids, especially lycopene and beta-carotene. Phenolic compounds, such as gallic acid, protocatechuic acid, *p*-hydroxybenzoic acid, chlorogenic acid, caffeic acid, syringic acid, *p*-coumaric acid, ferulic acid, and sinapic acid, are also indicated in the aril part. In addition, the flavonoid compounds rutin and luteolin were also abundant in the aril part [15]. The aril extract demonstrated interesting functions *in vitro*. For example, aril extract was found to reduce the oxidative stress from LPS-induced reactive oxygen species (ROS), demonstrating antioxidant activity [15]. In addition, inflammation from LPS-induced macrophage raw 264.7 cells was suppressed by aril extract via regulation of NO, iNOS, COX-2, TNF-*α*, and IL-6 production [16]. Aril extract also demonstrated antimelanogenesis activity by reducing tyrosinase activity and p-PKC expression [17]. Moreover, the activity of aril prevented lipid accumulation in HFD-induced adiposity [18]. Aside from the activities mentioned, aril extract has also been shown to suppress the cell proliferation and angiogenesis of human colon 26–20 cells and HepG2 cells [19]. Markedly, aril extract has been shown to inhibit the growth of breast cancer cells and melanoma [12, 20]. Although several mechanisms of action have been studied, the mechanism of cancer metastasis has not yet been investigated.

Cancer metastasis is a key process in cancer progression and consists of several steps, including cell proliferation, migration, invasion, adhesion, and angiogenesis [21]. To regulate metastasis, many protein mediators and signaling events are needed. Of these mediators, matrix metalloproteinases (MMPs) have been shown to be significant for cancer cells to migrate and invade other tissues. MMPs are zinc-dependent endopeptidase enzymes that function to degrade the extracellular matrix [22]. This degradation allows cancer cells to evade the primary sites and easily move through other organs. Overexpression of MMPs has been associated with aggressive malignant tumors and poor prognosis in patients [23]. Presently, 28 MMP members are indicated in a family. However, MMP members that have a major role in cancer cell migration and invasion include MMP-2 and MMP-9 [24]. MMP-9 participates in tumor metastasis in several tumor cancers, including lung cancer [25], cervical cancer [26], and prostate cancer [27]. Thus, the suppression of MMP function is considered a crucial targeted therapy for cancer treatment [28].

Therefore, we hypothesized that the aril extract from gac fruit may have promising activity by suppressing prostate cancer cell proliferation and metastasis. We used highly metastatic prostate cancer cells (PC-3) as a model compared with low metastatic prostate cancer cells (LNCaP). The process of cancer cell migration and invasion was determined to observe whether aril extract could suppress metastasis. Additionally, signaling through MMP-9 and TIMP-1 was investigated. As shown in our results, aril extract could suppress prostate cancer cell proliferation in both high- and low-metastasis models and was not toxic to normal Vero cells. Importantly, aril extract demonstrated the suppression of PC-3 migration. Additionally, the expression of MMP-9 was reduced, while TIMP-1 was increased. These data indicated a novel function of aril extract and provided the value of gac fruit as a potential source to develop as an antimetastatic agent for prostate cancer patients.

## 2. Materials and Methods

### 2.1. Reagents

Roswell Park Memorial Institute (RPMI) 1640 medium, Dulbecco's modified Eagle (DMEM), Eagle's minimum essential medium (EMEM), fetal bovine serum (FBS), and penicillin/streptomycin were purchased from Gibco (Thermo Fisher Scientific, Inc., Waltham, MA, USA). 3-(4,5-Dimethylthiazol-2-yl)-2,5-diphenyltetrazolium bromide (MTT), dimethyl sulfoxide (DMSO), propidium iodide (PI), trypan blue, trihydroxy benzoic acid (Gallic acids), and Folin–Ciocalteu's reagent were purchased from Sigma (St. Louis, MO, USA). All the plates used in this study were purchased from Corning (Corning, NY, USA). Ethanol, acetone, and hexane were purchased from RCI Labscan (Bangkok, Thai-land). Ammonium persulfate, tetramethyl ethylenediamine (TEMED), protein marker, and acrylamide were purchased from Bio-Rad (California, USA). Tris base was purchased from Vivantis (Selangor Darul Ehsan, Malaysia).

### 2.2. Cell Culture

Human prostate carcinoma cells (PC-3 and LNCaP) were purchased from American Type Culture Collection (ATCC, Manassas, VA, USA). PC-3 has high metastatic potential, whereas LNCaP has low metastatic potential. PC-3 cells were maintained as monolayers in DMEM supplemented with 10% fetal bovine serum (FBS), 1% streptomycin–penicillin, and 3.7 g/L sodium bicarbonate (NaHCO_3_) as a pH buffer agent. LNCaP cells were maintained in RPMI 1640 containing 10% FBS, 0.1% streptomycin-penicillin, 1 mM sodium pyruvate, 10 mM HEPES, and 2 g/L sodium bicarbonate (NaHCO_3_). Vero cells, an African green monkey kidney cell line (ATCC® CCL-81™), were purchased from ATCC and used as a representative normal cell line. Vero cells were cultured in EMEM containing 10% FBS and 1% penicillin/streptomycin. All cell types were grown in a 75 cm^3^ T flask (Corning, NY, USA) under 95% humidified air with a 5% carbon dioxide (CO_2_) incubator at 37°C.

### 2.3. Preparation of Different Extracts from Gac Aril

Gac fruits soon after harvest were collected from Nakhon Pathom Province, Thailand (red color). The fruits were cleaned with 70% alcohol for 10 min to reduce any possible contamination. The arils part of gac fruits was separated and stored at −80°C in the dark condition until subsequent experiments. To prepare crude extracts, the aril sample (4 g) was soaked with a different type of solvent (100 ml). Four extracts were prepared as follows: (1) soaked aril sample in the water and boiled for 20 min, (2) soaked aril sample in the water and ultrasonicated for 30 min at 50 Hz, (3) soaked aril sample with 80% EtOH for 24 h, and (4) soaked aril sample with hexane : acetone : ethanol (H : A : E, 2 : 1 : 1) for 24 h. Afterward, each extract was filtered through Whatman paper No 1. Then, boiled and sonicated extracts were lyophilized at −110°C using a lyophilizer machine (Scanvac, SPC RT CO., Thailand) and dissolved with MilliQ water before being used in the experiments while EtOH and HAE extracts were evaporated at 40°C and dissolved in DMSO before being used in the experiments.

### 2.4. MTT Assay

The effects of different aril extracts on the viability of PC-3, LNCaP, and Vero cells were measured using an MTT assay. Briefly, 1 × 10^4^, 2 × 10^4^, and 1 × 10^4^ cells/well of PC-3, LNCaP, and Vero cells, respectively, were seeded in 96-well culture plates with DMEM, RPMI 1640, and EMEM medium containing 10% FBS and antibiotics overnight to reach 80% confluency. The media were replaced with fresh medium containing different aril extracts at various concentrations (0–5 mg/mL) and cultured at 37°C in 5% CO_2_ and 95% air for 24, 48, and 72 h. The medium with 0.5% DMSO was used as a vehicle control. After treatment, 10 *μ*l of 5 mg/mL MTT solution was added to each well and cultured further for 4 h. Then, 100 *μ*L of DMSO was added to each well to dissolve purple formazan crystals. The absorbance of the samples was determined at a wavelength of 570 nm using a multimode microplate reader (Synergy; BioTek Instruments, Inc., Santa Clara, CA, USA).

### 2.5. Determination of Total Phenolic Content

The total phenolic content of the sonicated extract was measured according to the Folin–Ciocalteu method [23]. Briefly, 25 *μ*L of extract (1 mg/mL prepared in methanol) was mixed with 87.5 *μ*L of 10% Folin–Ciocalteu reagent in 96-well plates and incubated for 5 min. Following incubation, 87.5 *μ*L of 6% Na_2_CO_3_ was added, and the plate was mixed and incubated in the dark for 1.5 h. Then, wells were read at 725 nm using a microplate reader (Thermo Fisher Scientific, Waltham, MA, USA). The total phenolic content was quantified using a gallic acid standard curve.

### 2.6. Lactate Dehydrogenase (LDH) Cell Damage Assay

Cell damage was measured using an LDH assay kit, following the manufacturer's instructions (G-Biosciences, St. Louis, MO). PC-3 cells were seeded in 96-well plates at a density of 1 × 10^4^ cells/well and allowed to adhere overnight. Next, the cells were treated with sonicated extracts at 0–10 mg/mL for 24 h. Then, the supernatants (25 *μ*L) were transferred to a new 96-well plate, and 25 *μ*L of LDH reaction mixture was added to each well. All reactions were incubated for 20 min at 37°C in the dark. The LDH release was analyzed using a multimode microplate reader (Synergy; BioTek Instruments, Inc., Santa Clara, CA, USA) at 490 nm.

### 2.7. Cell Cycle Distribution

The cell cycle distribution was investigated using propidium iodide (PI) staining and evaluated with a flow cytometer. PC-3 cells were plated in 6-well plates at a density of 3 × 10^5^ cells per well and allowed to adhere overnight. Then, the cells were treated with sonicated extract at 0, 2, 4, 6, and 8 mg/mL. After 24 h of treatment, the cells were harvested and fixed in 70% chill-EtOH overnight at 4°C. Next, the cells were centrifuged at 1,800 rpm for 5 min to remove EtOH and washed twice with PBS. The cells were stained with PI (50 *μ*g/mL) and RNase (20 *μ*g/mL) for 30 min and analyzed. The percentage of DNA content in each phase was determined by using a Guava EasyCyte™ flow cytometer and GuavaSoft™ software (Merck Millipore Crop., Merck KGaA).

### 2.8. Wound Healing Assay

The wound healing assay was performed to test the cell migration ability of PC-3 cells when incubated with the sonicated extract. PC-3 cells were seeded in 6-well plates at a density of 5 × 10^5^ cells/well in serum medium and incubated until 90% confluence. The cell monolayer was scratched with a sterile 200 *μ*L pipette tip. Subsequently, the cells were washed twice with PBS to remove cell debris and then treated with nontoxic concentrations of sonicated extract (2, 4, and 6 mg/mL) in fresh medium. A series of images along the scratch were randomly taken at 0 and 24 h using an Olympus IX70 inverted microscope. Areas of the wounds were measured in three independent groups using ImageJ software (Java 1.8.0_112 (64_bit)), and relative cell migration was calculated as the wound area at 24 h.

### 2.9. Transwell Migration Assay

PC-3 cell migration was performed as described by Kou et al. [27]. Briefly, PC-3 cells (4 × 10^4^) were resuspended in 200 *μ*L of serum-free medium with or without 2, 4, or 6 mg/mL sonicated extract and placed on the upper well of a transwell insert with 8 *μ*m pores (Corning, MA, US). Then, 10% FBS-containing medium (DMEM) (600 *μ*l) was added to the lower chamber and incubated for 24 h at 37°C to allow cell migration to the lower chamber. To measure the migrated cells, the bottom of the upper wells was fixed with 100% methanol at room temperature (RT) for 20 min, stained with 0.5% crystal violet at RT for 15 min, and washed with PBS several times until all the excess dye had been removed. The cells that did not migrate from the upper chamber were removed using cotton swabs. The cells that migrated through the pores of the insert into the lower chamber were photographed under a light inverted microscope at a magnification of ×10. Finally, the optical density of the membrane with the migrated cells was measured at a wavelength of 570 nm. Data are presented as the means ± SEM (*n* = 3).

### 2.10. Western Blot Analysis

PC-3 cells were seeded in a 6-well plate at a density of 3 × 10^5^ cells per well and allowed to adhere for 24 h. Afterward, the cells were replaced with fresh medium containing the sonicated extract at 0, 2, 4, and 6 mg/mL for 24 h. Then, the cells were harvested, and protein contents were extracted using RIPA buffer (1 M Tris-HCl pH 7.4, 5 M NaCl, 20% NP-40, 10% sodium deoxycholate, 20% SDS, and a protease inhibitor cocktail). Extracted proteins were kept at −80°C for further use. The protein concentration was determined using the Bradford assay. Equal amounts of proteins were loaded and separated by sodium dodecyl sulfate–polyacrylamide gel electrophoresis (SDS–PAGE) (Bio-Rad Laboratories, Hercules, CA, USA) at 100 V (initial setting at 55 V for 20 min) for 1.5 h at room temperature and then transferred to a PVDF membrane. The membranes were rinsed in TBST and blocked with 5% BSA in TBST for 1 h at room temperature. Then, the membrane was incubated with the primary antibodies (anti-MMP-9, anti-TIMP-1, and anti-GAPDH) in fresh 3% BSA at 4°C overnight. The antibody-bound membranes were washed 3 times in TBST, each for 10 min. They were then treated with the specific anti-rabbit secondary antibody in 3% BSA and incubated for 1 h at room temperature on a slow shaker followed by washing 3 times. The immunoreactive signals were detected with ECL plus™ chemiluminescent substrate (Bio-Rad Laboratories, Hercules, CA, USA) and detected by a gel documentary machine (UVITEC, Alliance Q9 Advanced, Cambridge, UK). The band intensity of proteins was quantified by ImageJ software [Java 1.8.0_112 (64_bit)].

### 2.11. Statistical Analysis

All data generated are presented as the means ± SEM of three independent experiments unless otherwise specified and were analyzed using GraphPad Prism 5.03 software. Multiple comparison analyses were performed using one-way ANOVA (analysis of variance) followed by Dunnett's postmultiple comparisons test. *p* < 0.05 was considered to indicate a statistically significant difference. All experiments were carried out in triplicate.

## 3. Results

### 3.1. The Effect of Aril Extracts on Cell Proliferation of Prostate Cancer and Normal Cells

**Figure 1:**
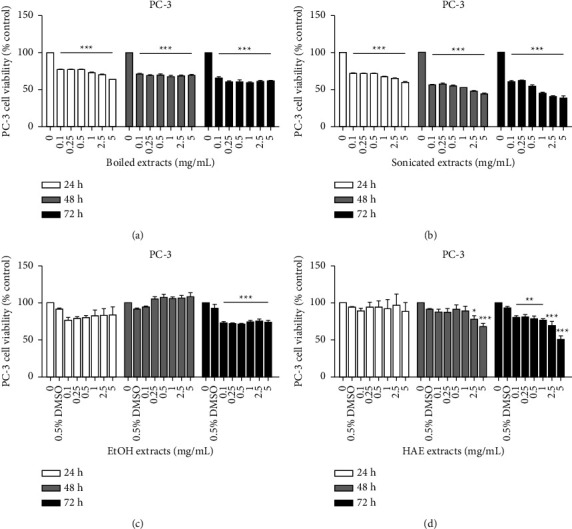
The antiproliferative effect of crude aril extracts in PC-3 cells. Cells were cultured in 96-well plates and treated with various concentrations of different crude extracts (0–5 mg/mL). (a) Boiled extract, (b) sonicated extract, (c) EtOH extract, and (d) HAE extract, and incubated for 24, 48, and 72 h. Cell viability was determined by MTT assay. Data are presented as the means ± SEM (*n* = 3) (*p* < 0.05^*∗*^, *p* < 0.01^*∗∗*^, and *p* < 0.001^*∗∗∗*^ were considered significant compared to control) of three independent experiments.

**Figure 2:**
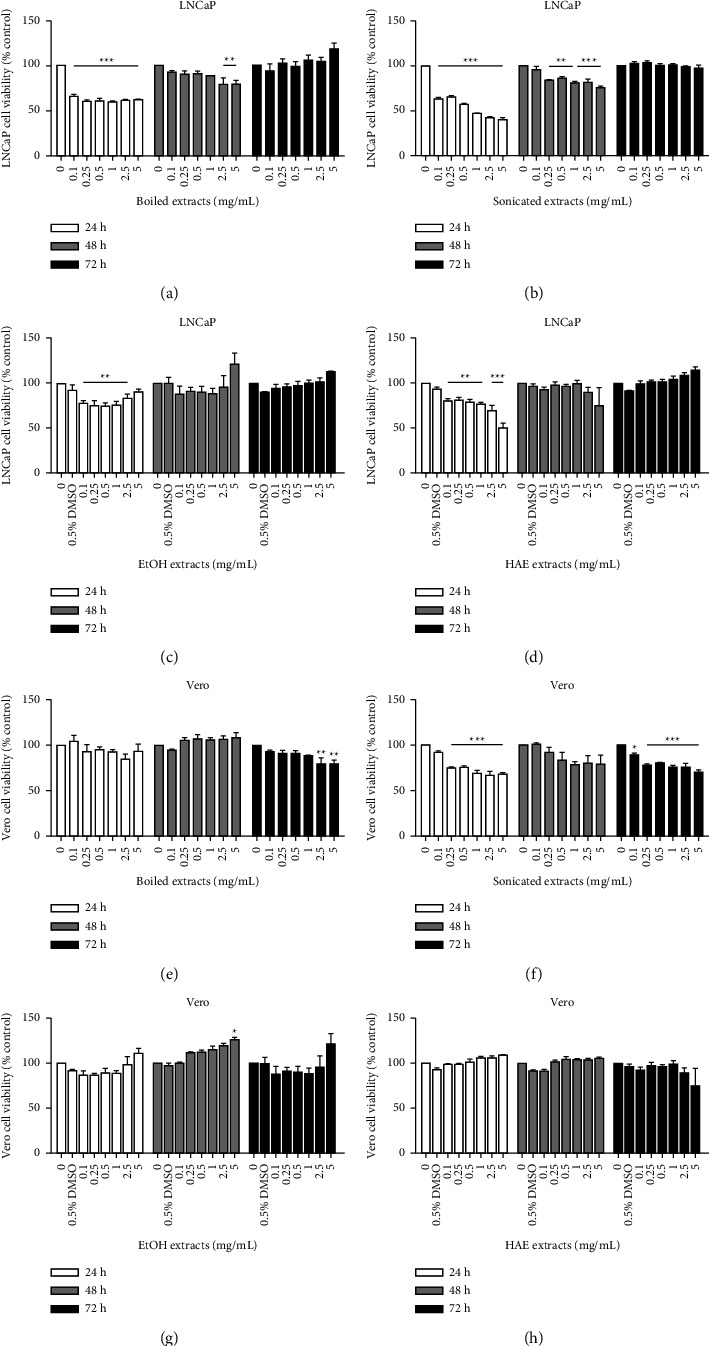
The antiproliferative effect of various crude extracts from aril parts: (a–d) LNCaP cells; (e–h) Vero cells. (a–h) Cells were cultured in 96-well plates, treated with various concentrations of different extracts (0–5 mg/mL), and incubated for 24, 48, and 72 h, respectively. The cell viability of each extract was determined by MTT assay. Data are presented as the means ± SEM (*n* = 3) (*p* < 0.05^*∗*^, *p* < 0.01^*∗∗*^, and *p* < 0.001^*∗∗∗*^ were considered significant compared to the control) of three independent experiments.

To observe the inhibitory effect of gac aril on prostate cancer cell proliferation, different aril extracts were screened in PC-3 (high-aggressive) and LNCaP (low-aggressive) cells and compared with normal Vero cells (African green monkey kidney) using an MTT assay. The crude aril extracts were prepared with different solvents and methods, including boiling in water, sonication in water, soaking in 80% EtOH, and soaking in H : A : E (2 : 1 : 1) solvent. The maximum final concentration of DMSO (0.5%) was used as the vehicle control. All cells were treated with 0–5 mg/mL of each aril extract for 24, 48, and 72 h. As shown in Figures 1 and 2, the sonicated extract clearly inhibited prostate cancer cell proliferation both in PC-3 and LNCaP cells but less affected Vero cells. The IC_50_ values for PC-3 cells were 2.01 ± 0.02 and 0.59 ± 0.02 mg/mL at 48 and 72 h, respectively (Figure 1(b)), while for LNCaP, they were 0.56 ± 0.03 mg/mL at 24 h (Figure 2(b)). Both the boiled extract and ethanol extract had no inhibitory effect on prostate cancer cell proliferation. Meanwhile, the HAE extract affected PC-3 cells at 72 h, of which the IC_50_ was 6.04 ± 0.12 mg/mL. For LNCaP, HAE was affected only at 24 h, and the IC_50_ value was 6.25 ± 0.04 mg/mL. These results indicated that the aril extract prepared from sonication had the highest efficiency and thus was selected for further study of the antimetastatic mechanism.

### 3.2. The Sonicated Extract Did Not Induce PC-3 Cell Damage or Death

**Figure 3:**
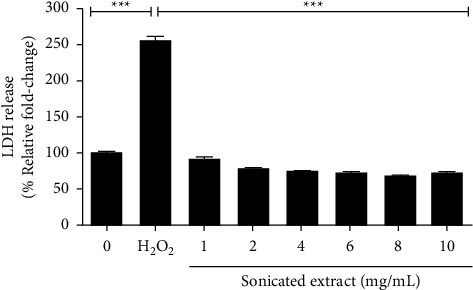
The cell damage effect of sonicated extract on PC-3 cells. PC-3 cells were treated with sonicated extracts and incubated for 24 h, and LDH released into the media was measured at 490 nm using a microplate reader. The graph presents the LDH release in medium collected from sample groups. Data are presented as the means ± SEM (*n* = 3). (*p* < 0.001^*∗∗∗*^ was considered significant compared between the negative control and positive group, positive and treated control) of three independent experiments.

In a further experiment, an LDH assay was used to test whether sonicated extract causes damage to PC-3 cells. As shown in Figure 3, the range of concentrations of sonicated extract from 1–10 mg/mL did not induce PC-3 cell damage. The LDH levels were the same as those of a negative untreated control sample. H_2_O_2_ was used as a positive control and clearly showed a high LDH release. These results suggested that the sonicated extract did not damage prostate cancer cells; however, it affected their proliferation.

### 3.3. The Sonicated Extract Might Attenuate the DNA Synthesis Process on PC-3 Cells

**Figure 4:**
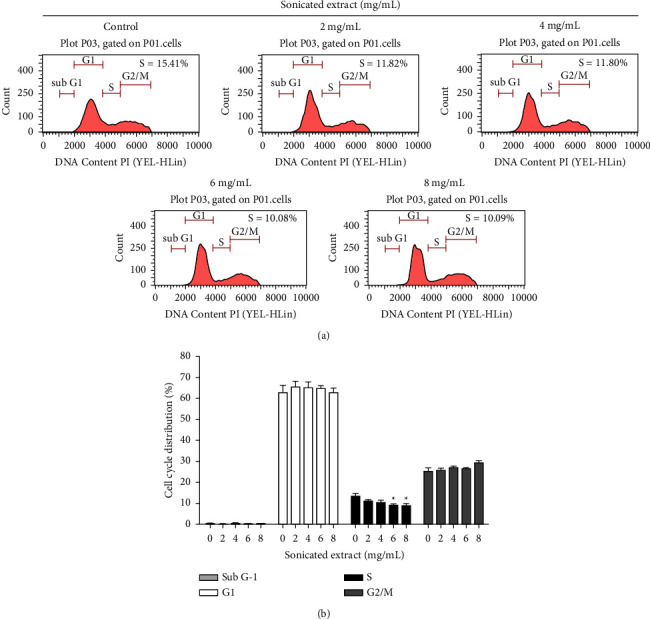
Cell cycle analysis in prostate cancer cells treated with sonicated extract. (a) PC-3 cells were treated with sonicated extract for 24 h, and cell cycle analysis was performed by flow cytometry using propidium iodide (PI) staining. (b) The percentages of cells in sub-G1, G1, S, and G2/M phases are indicated. Data are presented as the means ± SEM (*n* = 3). *p* < 0.05^*∗*^ was compared with the control group by one-way ANOVA (Dunnett's test).

To confirm that sonicated extract did not cause cell damage, a cell cycle analysis was performed. Propidium iodide was used for DNA staining, and the amount of DNA content in each cell cycle was determined using a flow cytometer. As shown in Figures 4(a) and 4(b), the sonicated extract at 0, 2, 4, 6, and 8 mg/mL could not induce cancer cell death. The sub-G1 or apoptotic populations were not detected in all treated samples. The number of populations of the G1 and G2/M phases was in the same range in all treated samples. However, the population of the S phase was significantly reduced. The values were 11.82%, 11.80%, 10.08%, and 10.09% at 2, 4, 6, and 8 mg/mL, respectively. These results indicated that the sonicated extract did not cause cell death but may interfere with the cancer cell cycle at the S phase.

### 3.4. The Sonicated Extract Inhibits PC-3 Cell Migration

**Figure 5:**
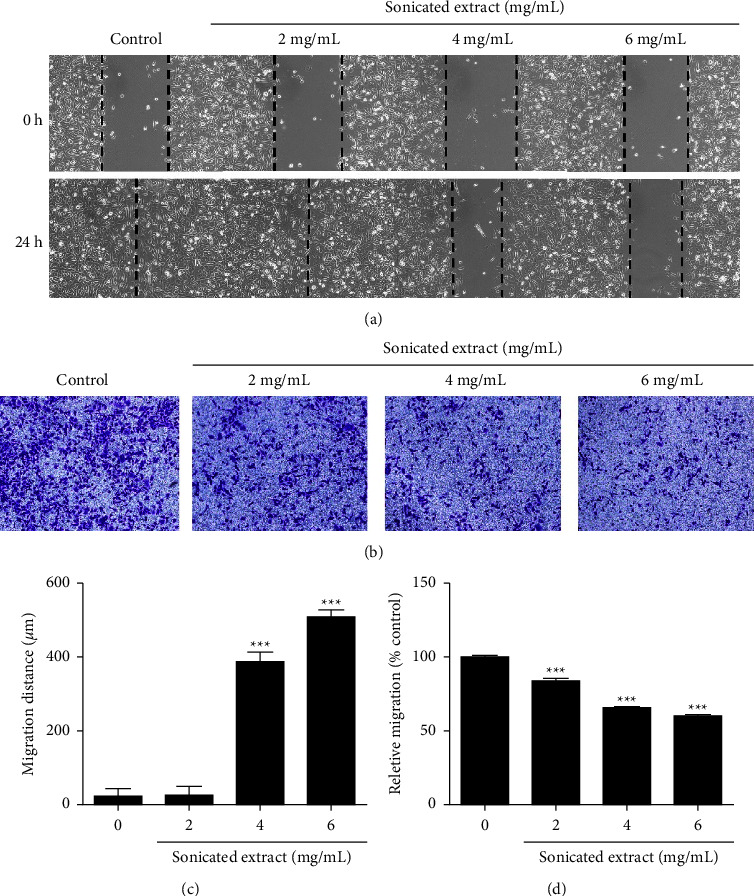
Inhibitory effect of the sonicated extract on PC-3 cell migration. PC-3 cells were treated with sonicated extract at 0, 2, 4, and 6 mg/mL and incubated for 24 h. (a) The migration of PC-3 cells was measured using a wound healing assay. After 0 and 24 h of migration, the scratches were photographed. (b) The migration of PC-3 cells was measured using the transwell insert migration assay. (c) The migration distance was calculated. (d) The relative migration of PC-3 cells was determined as described in the methods. All experiments were repeated three times. Data are presented as the means ± SEM (*n* = 3), *p* < 0.01^*∗∗*^, *p* < 0.01^*∗∗∗*^ versus the control group.

To determine the effect of the sonicated extract on prostate cancer cell metastasis, the process of cancer cell migration was chosen as a target for investigation. A wound healing assay was performed by treating PC-3 cells with the sonicated extracts at 0, 2, 4, and 6 mg/mL for 24 h. As shown in Figures 5(a) and 5(c), a relative wound recovery of 100% was clearly shown in the untreated control sample and in the 2 mg/mL sonicated extract-treated sample. Meanwhile, at 4 and 6 mg/mL, the percentage of wound recovery was significantly reduced to less than 50% compared to the untreated control. These results revealed that sonicated extract significantly inhibited prostate cancer cell migration. The ability of the sonicated extract to suppress the migration of PC-3 prostate cancer cells was further confirmed using a transwell migration assay. The results are illustrated in Figures 5(b) and 5(d), showing that PC-3 cells that migrated through the lower chamber were significantly reduced after they were treated with sonicated extracts in a dose-dependent manner.

### 3.5. The Sonicated Extract Affects the Expression of Protein Regulators Related to Cancer Cell Migration

**Figure 6:**
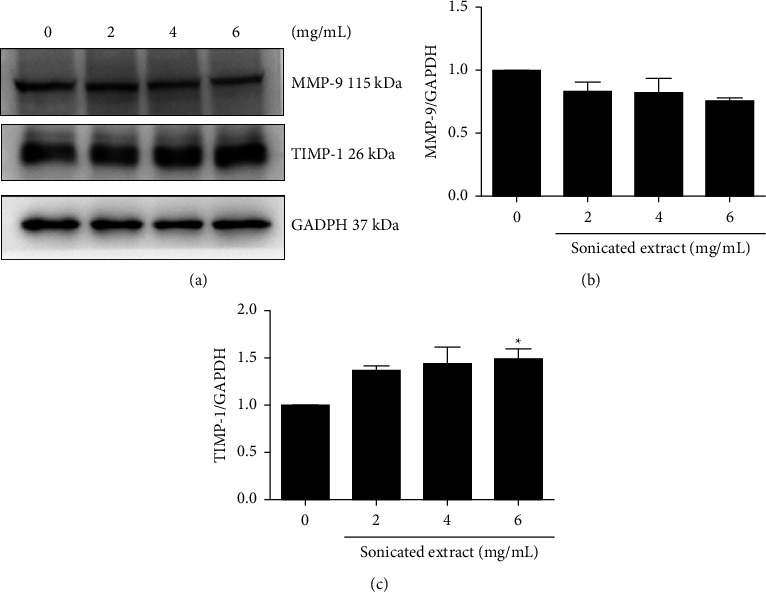
The effect of the sonicated extract on the expression of protein regulators in cancer cell migration. PC-3 cells were treated with the sonicated extracts at 0, 2, 4, and 6 mg/mL for 24 h. The protein expression levels of MMP-9 and TIMP-1 were evaluated using a Western blot assay. (a) The expression of MMP-9 and TIMP-1 normalized to the GAPDH loading control. (b–c) The relative band intensity was quantified by Image software. Data are presented as the means ± SEM (*n* = 3). *p* < 0.05^*∗*^ was compared with the control group by one-way ANOVA (Dunnett's test).

To understand how sonicated extract suppresses prostate cancer cell migration, the expression of protein regulators was detected using western blotting. Matrix metalloproteinase-9 (MMP-9) was selected because it is an important enzyme used in prostate cancer cell migration and invasion. Tissue inhibitor of metalloproteinase-1 (TIMP-1), an inhibitor of MMP, was also selected. PC-3 cells were treated with 0, 2, 4, and 6 mg/mL sonicated extract for 24 h. The results showed that sonicated extract significantly decreased MMP-9 expression compared with the untreated control (Figures 6(a) and 6(b)). The expression level of TIMP-1 was increased in the sonicated extract-treated groups (Figure 6(c)). These results indicated that sonicated extract suppresses the process of prostate cancer cell migration through the downregulation of MMP-9 and TIMP-1 upregulation.

### 3.6. Phenolic Compounds as One Major Component of the Sonicated Extract

To determine the number of phenolic compounds, which are secondary metabolites that are mostly found in plants and responsible for plant biological function, the total phenolic content was evaluated in the sonicated extract. As a result, the sonicated extract from gac aril provided 19.99 ± 1.86 mg GAE/g. These results indicated that sonicated extract consists of phenolic compound sources that might affect cell proliferation and/or metastasis processes.

## 4. Discussion

Prostate cancer is a common cancer found in men that is highly treatable if diagnosed in the early stage. However, many low- and middle-income countries still have a problem with screening and early detection, which cause a high number of deaths from metastatic prostate cancer. Thus, novel efficient treatments, as well as effective therapeutic agents that can overcome metastasis, are needed. Since natural substances from vegetables and fruits are considered valuable sources for anticancer drug development, *M. cochinchinensis* or gac fruit was selected in this work. Gac has been reported to have various biological activities, including antioxidant [15], anti-inflammatory, and anticancer activities [14, 16]. Several parts of gac are indicated for these activities, including the seeds, stems, leaves, roots, and arils [13]. We showed further in this work that aril could inhibit prostate cancer cell proliferation and migration.

The investigation was performed in both high-metastasis (PC-3) and low-metastasis (LNCaP) human prostate cancer cells compared with Vero cells. The gac aril was prepared with different methods and solvents to obtain different active components. Of the four extracts, boiled extract, sonicated extract, EtOH extract, and HAE extract, the antiproliferative effect against PC-3 and LNCaP cells indicated that sonicated extract gave the highest efficiency. Τhe IC_50_ values of sonicated extract for PC-3 were 2.01 ± 0.02 mg/mL and 0.59 ± 0.02 mg/mL for 48 and 72 h, respectively. The antiproliferative levels of the sonicated extract are in the same range as shown in Wimalasiri's report that the IC_50_ values of water extract from gac aril for human melanoma cancer cell lines (MM418C1 and D24) were 0.49 mg/mL and 0.73 mg/mL, respectively [12]. The effect of sonicated extract inhibits only the proliferation process and does not damage or cause cancer cell death. As depicted in Figure 3, 1–10 mg/mL of the sonicated extract did not induce LDH release when compared with H_2_O_2_, a positive control. LDH is a cytosolic enzyme that is usually used as an indicator of cell death or cytotoxicity. It is rapidly released into the cell culture medium when cells are damaged [29]. Cell cycle analysis also confirmed the antiproliferative effect of the sonicated extract, as an undetectable sub-G1 peak or apoptotic population was found, while a reduction in S-phase was detected (Figures 4(a) and 4(b)). These data illustrate that the sonicated extract may interfere with the process of DNA synthesis or replication. In addition, the effect of the sonicated extract was selectively acted on prostate cancer cells and less on normal cells. As depicted in Figure 2(f), the sonicated extract reduced the proliferation of Vero cells but to a lesser extent than in PC-3 cells (Figure 1(b)). At 5 mg/mL, the sonicated extract reduced the proliferation of Vero cells by approximately 30%, while in PC-3 cells was less than 50%. This selective effect is possibly from the different properties between cancer and normal cells. Cancer cells can quickly grow and proliferate as unlimiting compared to normal cells [30]. Overexpression of proteins involving cell cycle progression and apoptosis inhibition is highly found in many cancer cell types [31–35]. Most anticancer agents selectively targeted these proteins, especially from natural origin. For example, the anticancer effect of curcumin has been indicated selectively induces colon cancer cell apoptosis and S cell cycle arrest by regulating Rb/E2F/p53 pathway. A selective effect has been indicated from the difference in the DNA repair system which is more efficient in normal cells than transformed cells [36]. The effect of isothiocyanates derived from Brassica plants comparing PC-3 prostate cancer cells and HDFa normal fibroblasts showing that the level of *γ*H2A.X, a marker of double-stranded DNA breaks, reduced more efficiently than in normal cells which indicated the activation of DNA repairing machinery [37].

The sonicated extract from gac aril also demonstrated an antimetastatic function in prostate cancer cells. As shown in Figure 5, the concentration of sonicated extract at 4–6 mg/mL significantly reduced the migratory ability of PC-3 cells, in accordance with the results of the transwell migration assay. The ability of cancer cell migration is a significant factor in cancer metastasis in which several mediators are involved. A crucial one is a group of matrix metalloproteinases (MMPs), which are proteolytic enzymes that are considered key mediators that enhance cancer progression. They degrade the extracellular matrix (ECM) and basement membrane, which facilitate the migration and invasion of cancer cells [38]. Increased expression of MMPs has been associated with a poor prognosis of aggressive cancer, especially MMP-2 and MMP-9 [39]. MMP-2 and MMP-9 belong to gelatinase, which is one of five groups of the MMP family. MMP-2 is released by cancer cells and can specifically degrade collagen type IV. MMP-9 enhances the metastasis of cancer cells by degrading collagen proteins of the ECM. To control the level of MMPs, other regulators called tissue inhibitors of metalloproteinases (TIMPs) are indicated. Indeed, Brehmer et al. show that the imbalance between MMPs and TIMPs results in the progression of metastasis in prostate cancer [40, 41]. As shown in Figure 6, PC-3 cells treated with the sonicated extract at 2, 4, and 6 mg/mL for 24 h demonstrated a reduction in MMP-9 expression, and this correlated well with an increase in TIMP-1, which normally binds specifically with MMP-9 [42]. Our results indicated that the sonicated extract suppresses prostate cancer cell migration via the regulation of MMP-9 and TIMP-1. These findings suggested that the sonicated extract from the aril part has the potential to act as an antimetastatic agent, which is a novel biological activity in this plant.

As indicated in the previous works, gac aril contains mainly phenolic and flavonoid compounds [15, 43]. Also, we found that the sonicated extract contains total phenolic compounds at 19.99 ± 1.86 mg GAE/g. This amount is much higher than previously reported in which the ethanolic extract of aril contains a total phenolic content of 4.29 ± 0.15 mg GAE/g. The phenolic compounds that have already been reported in gac aril were gallic acid, protocatechuic acid, *p*-hydroxybenzoic acid, chlorogenic acid, caffeic acid, syringic acid, *p*-coumaric acid, ferulic acid, sinapic acid [15]. All these compounds demonstrated either anticancer or antimetastatic activities [44–50]. Therefore, it is possible that phenolic compounds are active constituents of sonicated extract for suppression of prostate cancer cell viability and migration. This hypothesis is supported by many works showing that phenolic compounds from plants have anticancer and antimetastatic activities, which have been tested in several cancers, including skin cancer [44, 45], oral cancer [46], pancreatic cancer [47], breast cancer [51], lung cancer [52], prostate cancer [53], and brain tumor [54].

## 5. Conclusions

The present study demonstrated the value of *M. cochinchinensis* or gac fruit as an antimetastatic and anticancer agent. Gac aril extracted with sonication in water suppressed the proliferation of aggressive prostate cancer cells (PC-3). This effect did not interfere with normal Vero cells. Interestingly, the sonicated extract inhibited the migration of prostate cancer cells via the downregulation of MMP-9 and upregulation of TIMP-1. Hence, a sonicated extract from gac aril is worth studying further to add basic knowledge for anticancer drug development from plants.

## Data Availability

The authors confirm that the data supporting the findings of this study are available within the article.
